# Thermal and pH Responses of Hydrogels Based on Chitosan-*g*-*N*-Alkyl-Substituted Polyacrylamide Copolymers

**DOI:** 10.3390/polym18212787

**Published:** 2026-11-07

**Authors:** José Javier Coca-Hidalgo, Carlos Peniche-Covas, Jaime Lizardi-Mendoza, Waldo Manuel Argüelles-Monal

**Affiliations:** 1 Centro de Investigación en Alimentación y Desarrollo, Hermosillo 83304, Sonora, Mexico; jalim@ciad.mx; 2 Faculty of Chemistry, Universidad de La Habana, La Habana 10400, Cuba; peniche@fq.uh.cu

**Keywords:** chitosan-based copolymer hydrogels, poly(*N*,*N*-diethylacrylamide), poly(*N*,*N*-dimethylacrylamide), poly(*N*-ethylacrylamide), volume phase transition, hydrogen bonding

## Abstract

Chitosan grafted with *N*-alkyl-substituted polyacrylamide yields dual-responsive materials sensitive to both pH and temperature. For instance, the phase transition of poly(*N*,*N*-diethylacrylamide) can be tuned by copolymerizing it with more hydrophilic units, such as *N*,*N*-dimethylacrylamide or *N*-ethylacrylamide. While hydrophilic and hydrophobic interactions between macromolecular segments drive this phase transition, the specific role of hydrogen bonding between chitosan and the polyacrylamide grafts is not fully understood yet. Clarifying how these interactions influence the volume phase transition is essential for predicting hydrogel behavior. Chitosan-*g*-poly(DEAm-*co*-DMAm) and chitosan-*g*-poly(DEAm-*co*-NEAm) hydrogels showed continuous volume phase transitions that broadened with increased content of hydrophilic units (DMAm or NEAm). The critical temperature (*Tc*) of chitosan-*g*-poly(DEAm-*co*-DMAm) hydrogels increased linearly with the content of DMAm, while chitosan-*g*-poly(DEAm-*co*-NEAm) hydrogels exhibited a downward deviation from the linear variation in *Tc* from 0 to 100 mol-% DEAm. Both hydrogels showed higher swelling in acidic conditions at temperatures below the *Tc*, with a marked reduction as pH turned basic. Above the *Tc*, the pH response was almost negligible. Evidence suggests that the formation of hydrophobic domains of *N*,*N*-diethylacrylamide units during heating could bring the *N*-alkyl polyacrylamide and the chitosan chains closer together. This promotes inter-chain hydrogen bonding, which favors the cooperative character of the transition.

## 1. Introduction

Chitosan is a linear cationic polysaccharide, commonly obtained through the extensive deacetylation of chitin from residues of the crustacean processing industry. Its polymer chains are composed of acetylated and deacetylated units of glucosamine joined through β (1→4) linkages. The protonation of the amino groups in its structure makes chitosan soluble in acidic conditions and causes its behavior as a polyelectrolyte. However, the $-{NH}_{3}^{+}$ groups deprotonate in mid-alkaline conditions, causing a drop in charge density and solubility. The exceptional features of chitosan, including biocompatibility, biodegradability, gelification capacity, positive charge density at low pH, and antimicrobial properties, have led to widespread interest in this polysaccharide for commercial applications [1].

Grafting copolymerization techniques facilitate the integration of chitosan chains with other polymers to create materials with enhanced properties. In particular, the *grafting-on* approach has been widely used to polymerize *N*-alkyl-substituted polyacrylamides onto polysaccharide chains to prepare systems such as hydrogels with improved capabilities [2,3]. These hydrogels consist of three-dimensional, crosslinked polymer networks capable of absorbing large amounts of water and active substances within their porous structure. Their versatility has made them suitable for potential applications in the biomedical field [4], foods [5], or agriculture [6].

It is well established that poly(*N*,*N*-diethylacrylamide) undergoes a coil-to-globule transition in an aqueous solution, a process that is characterized by a lower critical solution temperature (LCST) [7]. When it forms a hydrogel, the transition becomes a volume phase transition, as the most evident thermal response is a change in gel swelling. Despite this fact, the cooperative character and the underlying interactions between the participating chains remain the same.

The balance of hydrophilic and hydrophobic interactions between macromolecular segments plays a major role during the thermal transition of poly(*N*,*N*-diethylacrylamide). These interactions and the critical temperature can be modulated by inserting more hydrophilic units into the poly(DEAm) chain. Copolymerization of DEAm has been successfully achieved with other hydrophilic acrylamide-derived monomers in previous work: *N*,*N*-dimethylacrylamide [8], acrylamide [9], *N*-isopropylacrylamide [10], and *N*-ethylacrylamide [11]. A lower number of carbon atoms in the *N*-alkyl substituent of the *N*-alkyl polyacrylamide has been related to higher hydrophilicity. Meanwhile, the NH moieties from the amide groups allow the monomer units to act as either a hydrogen-bond donor or acceptor with water molecules or polymer chains. However, there is no clarity about how the presence of hydrogen-bonding interactions between chitosan and the *N*-alkyl substituted polyacrylamides affects the volume phase transition in their hydrogels.

In this study, the thermal transition and the pH response of chitosan-*g*-poly(DEAm-*co*-DMAm) and chitosan-*g*-poly(DEAm-*co*-NEAm) hydrogels were characterized. The aim was to evaluate how varying the copolymer hydrophilicity—achieved by inserting two comonomer units with different hydrogen-bonding capacities: one an H-bond acceptor only and the other with both a donor and acceptor—affects the volume phase transition and functional properties of these hydrogels.

## 2. Materials and Methods

### 2.1. Materials

Low-molecular-weight chitosan was acquired from Sigma-Aldrich (Merck KGaA, Darmstadt, Germany) and used without further purification. Its molecular weight, M_V_ = 76,000, was estimated by viscometry [12], and the degree of deacetylation, DD = 84.5%, was determined by potentiometric and conductimetric methods.

The monomers *N*,*N*-diethylacrylamide, DEAm (Tokyo Chemical Industry Co., Tokyo, Japan); *N*,*N*-dimethylacrylamide, DMAm (Sigma-Aldrich, Merck KGaA, Darmstadt, Germany); and *N*-ethylacrylamide, NEAm (Sigma-Aldrich, Merck KGaA, Darmstadt, Germany) were purified by vacuum distillation. The crosslinker *N*,*N*-methylenbisacrylamide, MBA (Sigma-Aldrich, Darmstadt, Germany); ammonium persulfate, APS (Sigma-Aldrich, Darmstadt, Germany); *N*,*N*,*N*′,*N*′-tetramethylethylenediamine, TEMED (Sigma-Aldrich, Darmstadt, Germany); and methylene blue (Sigma-Aldrich, Darmstadt, Germany) were used as received. Type I ultrapure water with a conductivity lower than 0.05 μS cm^−1^ was used in all experiments.

### 2.2. Preparation of Chitosan-Based Hydrogels

The same hydrogel membranes used in the previous study were employed in this work [13]. The experimental procedure is briefly outlined as follows: Two series of copolymer hydrogels (chitosan-*g*-poly(DEAm-*co*-DMAm) and chitosan-*g*-poly(DEAm-*co*-NEAm)) were synthesized with different nominal compositions of comonomers. Briefly, a 2.0% *w*/*w* chitosan solution in 0.1 M HCl was prepared in a Schlenk tube and stirred overnight at room temperature. The corresponding amounts of both *N*-alkyl-acrylamide comonomers were successively dissolved in the chitosan solution along with the crosslinker, MBA, and the accelerator, TEMED. Quantities were calculated using the following ratios: comonomers:chitosan ≈ 5 g/g; APS = 0.7 mol-% of comonomers; TEMED:APS = 3 mol/mol; and MBA = 2.0 mol-% of comonomers. The mixture was degassed through four freeze–pump–thaw cycles under a high-purity argon atmosphere. The APS aqueous solution (c = 0.13 g/g) was added to the reaction mixture under agitation in an ice bath. The mixture was then transferred into 5 mL ampules, sealed with caps, and placed in a thermostatic bath at 20 °C for 24 h to polymerize. The obtained cylindrical hydrogels were cut into disk-shaped slices (14 mm diameter, 2 mm thick), dialyzed against water for a week with regular solvent changes, and dried at room temperature to a constant weight.

### 2.3. Swelling Measurements at Different Temperatures

The dried hydrogels were placed in thermally equilibrated (±0.02 °C) plastic containers filled with water (pH 6) for 12 h before determining the degree of swelling at the corresponding temperature. Once equilibrated, the hydrogels were removed from the water, carefully wiped to remove excess solvent, and then weighed. The degree of swelling, *W*, was calculated as:

(1) $$W=\frac{W_{i}-W_{0}}{W_{0}}$$

*W*_0_ and *W_i_* are the weights of hydrogels in a dry state and swollen at temperature *T*, respectively. Periodic measurements were carried out at selected temperatures from 20 to 85 °C. The experiments were conducted in triplicate, and the results were averaged for each data point (*W*).

### 2.4. Swelling Measurements at Different pH Levels

Dried, disk-shaped hydrogels of both chitosan-*g*-poly(DEAm-*co*-DMAm) and chitosan-*g*-poly(DEAm-*co*-NEAm) (50 mol-% composition) were weighed and allowed to swell in water (pH 6) at 10 ± 0.02 °C for at least 12 h. Meanwhile, pH-adjusted solutions ranging from pH 1 to pH 11 were prepared by diluting 1 M NaOH or HCl stock solutions.

The hydrogels swollen in water at 10 °C were transferred into the pH-adjusted solutions and allowed to reach equilibrium at 10 °C, and 50 or 70 °C for the copolymer containing NEAm or DMAm, respectively. After 24 h, their weights were measured at each temperature, as well as the pH value at the end of the experiment. The latter was the one taken into account in this study. The equilibrium swelling was calculated according to Equation (1).

### 2.5. Controlled Release Experiments

Encapsulating the model substance: Methylene blue (MB) was used to evaluate the ability to release a model substance under different conditions. The unloaded, dried, disk-shaped hydrogels (in triplicate) were immersed in 20 mL of an MB aqueous solution (C = 201 µg mL^−1^) at 10 °C for 24 h. The methylene blue-loaded hydrogels were rinsed with water to remove the excess dye and dried to a constant weight at room temperature.

*In vitro* release experiments: A plastic flask containing 75 mL of water (pH 6) as the release medium was immersed in a thermostatic bath at a fixed temperature. This volume is enough to keep the concentration stable at very low levels (lower than 2 × 10^−6^ M) and, therefore, to guarantee a stable gradient concentration throughout the study. After achieving thermal equilibrium, the loaded, dried, disk-shaped hydrogels (approximately 30 mg, 8.0 mm diameter, 0.8 mm thick each) were placed individually in the flasks. A 1 mL aliquot was collected periodically from the release medium and its absorbance measured at 665 nm using a UV spectrophotometer (UNICAM UV 500, Cambridge, UK). After the operation, the aliquot was returned to the flask. The solute concentrations were estimated using a calibration curve. Experiments were performed at temperatures below (10 °C) and above (50 °C) the thermal transition.

## 3. Results and Discussion

### 3.1. Preparation and Characterization of the Hydrogels

**Scheme 1:**
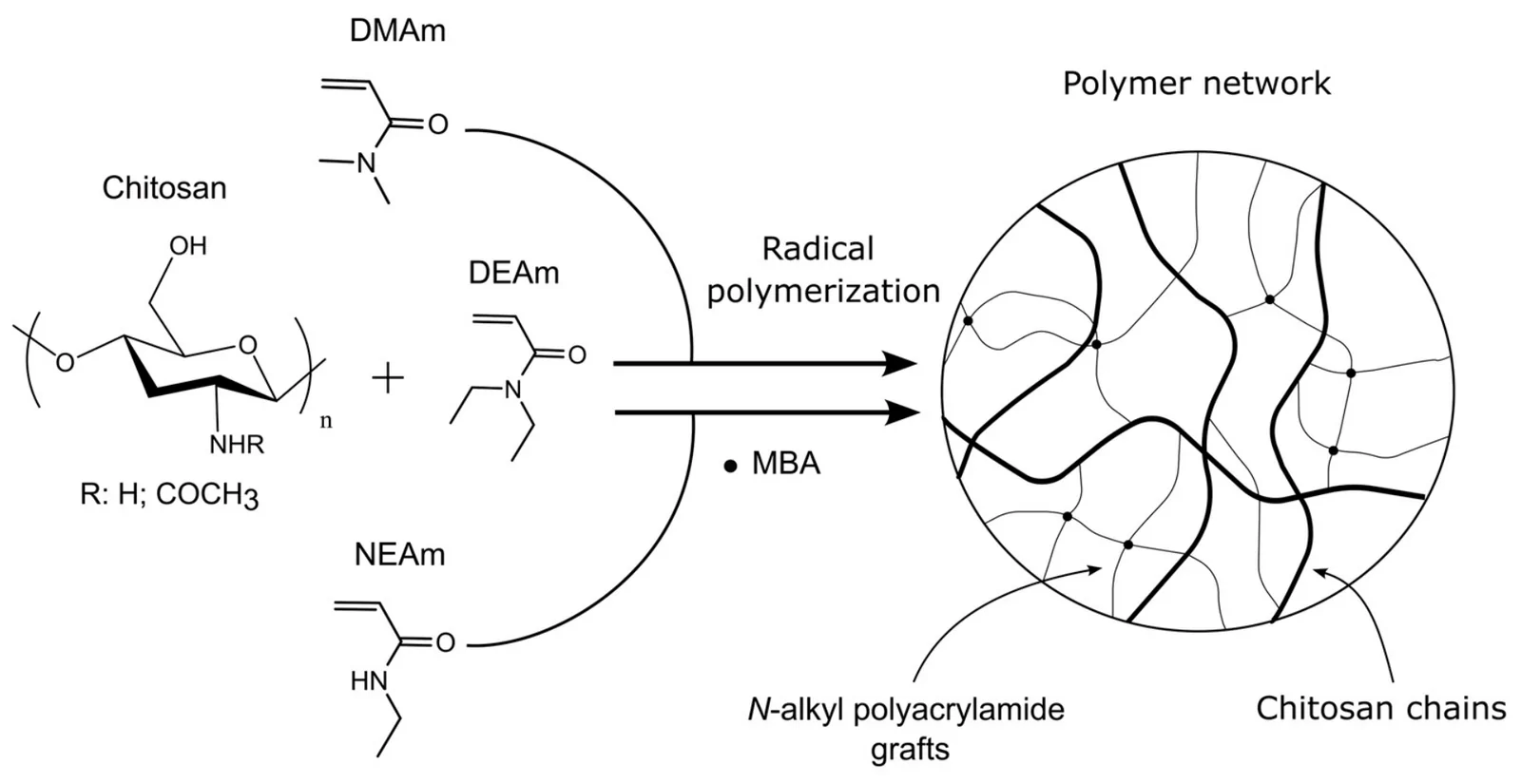
Synthesis of hydrogels based on chitosan grafted with *N*-alkyl polyacrylamide copolymers.

Hydrogels based on chitosan grafted with poly(DEAm-*co*-DMAm) and poly(DEAm-*co*-NEAm) were successfully prepared via radical polymerization at nominal compositions of 0, 25, 50, 75, and 100 mol-% DEAm, as depicted in Scheme 1.

Radical species generated from the homolytic decomposition of persulfate ions can react with chitosan chains, creating macro-radicals that can produce the grafted copolymer. A fixed amount of MBA was used to crosslink the newly formed chains to render a polymer network of chitosan macromolecules from which the chains of poly(DEAm-*co*-DMAm) and poly(DEAm-*co*-NEAm) are grafted.

The hydrogels produced through this method were strong and clear when swollen at low temperatures but turned opaque and contracted rapidly upon heating because of the thermal phase transition. It has been reported that these two copolymers have nearly the same composition as in the feed reaction mixture, with an almost random distribution along the polymer chain [11,14].

The characterization of these hydrogels was reported in a previous publication [13]. In summary, for both types of copolymers, the rheological measurements during polymerization showed the typical pattern of chemical gelation. Autoacceleration of the reaction was observed―more notable in the samples containing NEAm―possibly due to the high viscosity caused by the chitosan content, and the increased hydrogen-bonding capacity of NEAm units, which could slow down the termination reactions. The estimated gel times were also shorter in copolymers with NEAm units than the corresponding copolymers with DMAm units. The formation of strong gels was confirmed by measurements of the mechanical spectra, and their average pore sizes were estimated based on these measurements. The pore sizes ranged from 18 to 32 nm. The infrared spectra exhibited the characteristic bands of their respective *N*-alkyl-acrylamide components with band intensity varying according to the copolymer composition. All hydrogel thermograms showed three decomposition stages. The most important (from 200 to 450 °C), is related to the decomposition of the *N*-alkyl-substituted acrylamide groups with 80–90% weight loss (dry basis). The corresponding temperature of maximum decomposition rate varied from 400 to 420 °C, regardless of the copolymer composition. Based on time-dependent swelling data at 20 °C, water diffusion coefficients ranging from 1.08 to 2.8 × 10^−11^ m^2^ s^−1^, were estimated. In addition, the equilibrium swelling values increased as a function of NEAm content, while in hydrogels containing DMAm, they remained constant.

### 3.2. Thermal Response of Hydrogels

In order to study the thermal transition of chitosan-*g*-poly(DEAm-*co*-DMAm) and chitosan-*g*-poly(DEAm-*co*-NEAm) hydrogels, their swelling in water (pH 6) at different temperatures was first investigated. To this end, the hydrogels were first swollen in water at 20 °C for 12 h and subjected to a stepwise heating process; the swelling was then measured after equilibrium was reached at each temperature.

**Figure 1:**
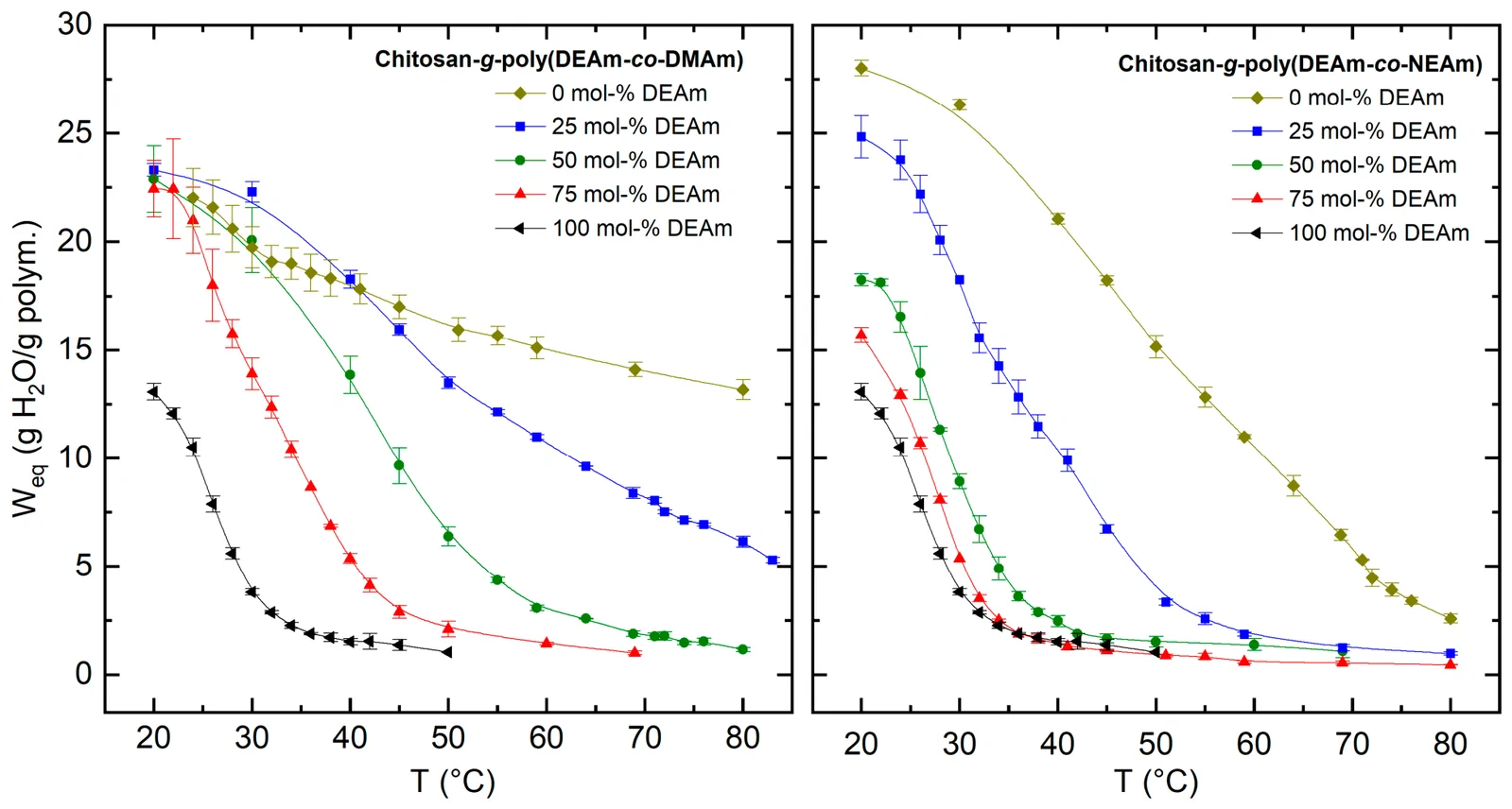
Effect of temperature on the equilibrium swelling of chitosan-*g*-poly(DEAm-*co*-DMAm) hydrogels (**left**) and chitosan-*g*-poly(DEAm-*co*-NEAm) hydrogels (**right**). Experiments were conducted in triplicate.

Figure 1 presents the equilibrium swelling curves for both series of chitosan-based hydrogels in the range of 20–80 °C. Each value represents the average of three replicates. Except for chitosan-*g*-poly(DMAm), all copolymers exhibited a remarkable decrease in volume change with increasing temperature, indicating a volume phase transition. For chitosan-*g*-poly(DMAm), a moderate, almost linear decrease in the swelling equilibrium values with heating is observed, although this hydrogel lacks a thermoresponsive component. Its behavior should be interpreted as a consequence of strengthening in the elastic retractile force of the polymer network as the temperature increases.

Conversely, the swelling curve of chitosan-*g*-poly(DEAm) decreases sharply, showing an inflection point at approximately 26 °C. This phenomenon resembles a first-order-like transition. In this case, an almost discontinuous change in gel swelling is observed during the transition from a swollen to a shrunken state when the temperature reaches the critical condition. At low temperatures, the mixing process is accompanied by unfavorable negative residual entropy, resulting from water ordering due to hydrophobic hydration. It keeps the polymer network in a swollen state. At temperatures above the critical temperature (*Tc*), the ordered water *icebergs* surrounding the hydrophobic groups disintegrate, causing water molecules to be expelled from the hydrogel, thereby increasing the entropy. Under these conditions, hydrophobic interactions between *N*-alkyl acrylamide groups dominate, leading to hydrogel shrinking.

Volume phase transitions are characterized by an unusual coexistence of swollen and contracted phases within the polymer network itself. The shape of the volume phase transition curve is the overall result of local phase changes that occur discontinuously within the polymer sample, each of which involves a polymer fraction that increases as the temperature rises. The main distinction is that the volume transition is an overall continuous process, which persists over a wide temperature range. This critical state exhibits several unusual and specific characteristics that depend on various factors ranging from the synthesis procedure to the method for measuring swelling, among others. In the words of Hirotsu, “the existence of this state itself violates the Gibbs phase rule” [15].

From Figure 1, it is also evident that as the content of the more hydrophilic comonomer in the copolymer increases, the thermal transition becomes broader, the traces are less abrupt, and the transition temperature shifts toward higher temperatures. These characteristics will be discussed in more detail later. Nevertheless, it cannot be ruled out that the increase in retractile elastic force with temperature may also influence the pattern of the swelling curves as a function of temperature.

A comparison of the volume phase transition in the two sets of copolymers was made by fitting the equilibrium swelling data points with a modified Boltzmann sigmoidal function [16]. It provides a simple way of calculating both the critical temperature and the slope of the volume phase transition, identifying the continuity of the phase transition. This model has been successfully validated using experimental data from various thermosensitive polymers, including *N*-alkyl polyacrylamides and polymethacrylates [16]. The model equation is:

(2) $$W=w_{f}-\frac{w_{f}-w_{i}}{\beta +e^{\frac{T-T_{c}}{\alpha }}}$$

where *w_i_* and *w_f_* represent the equilibrium values of the dependent variable before and after the transition, respectively. *T* is the independent variable and denotes the temperature corresponding to each point, while *Tc* is the critical volume phase transition temperature. The coefficient *α* quantifies the slope of the process during the transition and indicates whether the process is continuous or discontinuous. If *α* approaches 0, the phase transition becomes discontinuous as *T* → *Tc*. *β* is a parameter that determines the position of the critical value in relation to the geometric location of the transition. As *β* → 1, the critical temperature *Tc* approaches the geometric center of the transition. A non-linear least squares method was used to fit the experimental data to the model. In all cases, the ANOVA gave *p* values < 1 × 10^−4^.

**Figure 2:**
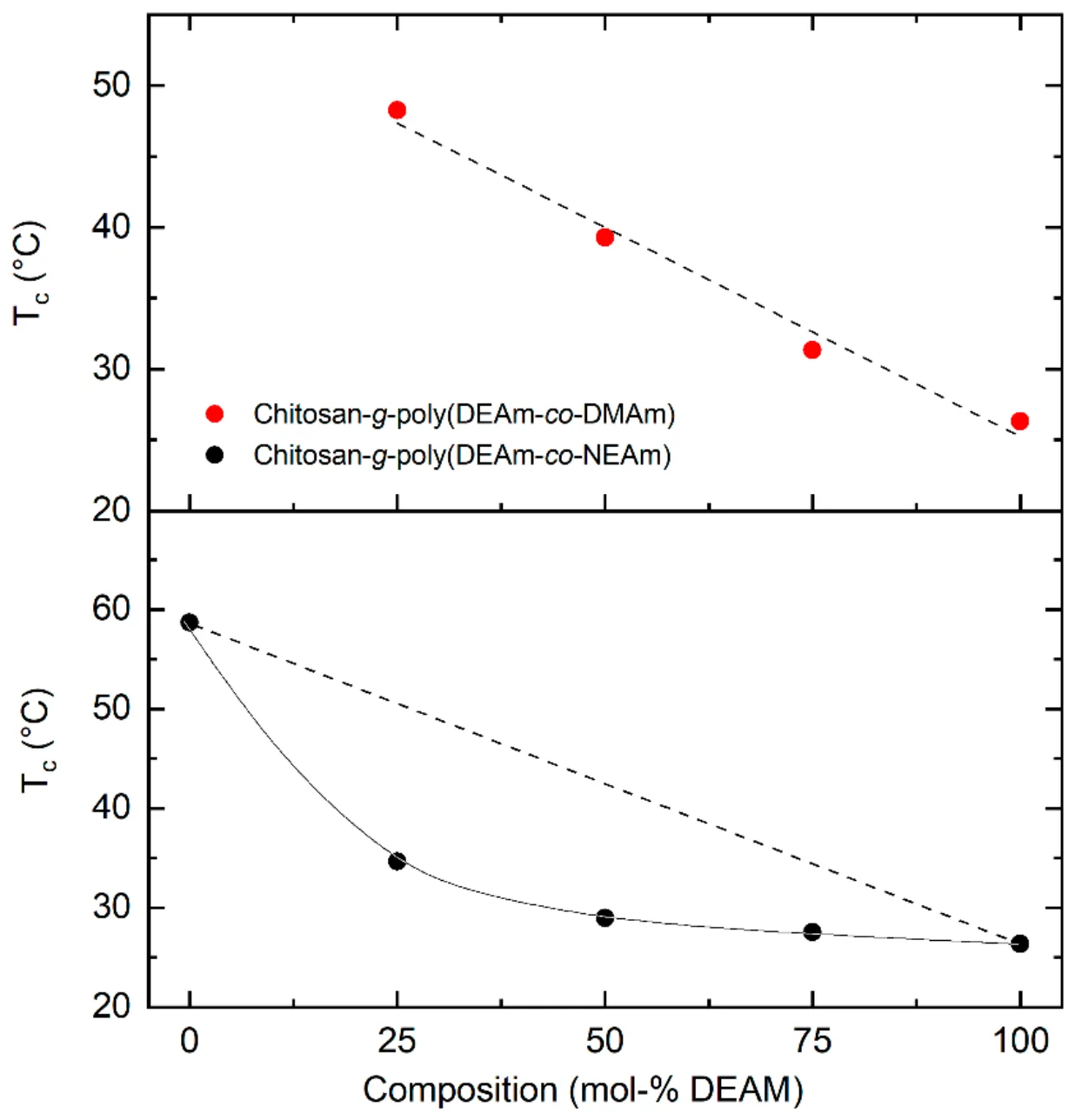
Effect of composition on the transition temperature, *Tc*, of chitosan-*g*-poly(DEAm-*co*-DMAm) (**top**) and chitosan-*g*-poly(DEAm-*co*-NEAm) hydrogels (**bottom**).

Figure 2 summarizes the critical temperatures for these two series of chitosan-based hydrogels as a function of DEAM content. In both systems, the critical temperature increases with the content of the more hydrophilic units (DMAm or NEAm). Specifically, for chitosan-*g*-poly(DEAm-*co*-DMAm), the *Tc* followed a linear relationship with composition. This dependence has been observed in other *N*-alkyl-disubstituted polyacrylamide copolymers [17]. The hydrogen-bonding potential of the tertiary amide nitrogen is due solely to the pair of lone electrons that can act as hydrogen-bond acceptors. However, this ability is weak due to the resonance of the amide group, which draws the nitrogen’s electron pair toward the carbonyl oxygen. Consequently, changes in the transition characteristics are primarily the result of the balance between the hydrophilic and hydrophobic units of the copolymers. This determines the linear variation in the transition temperature for this type of copolymer.

In contrast, the experimental values for chitosan-*g*-poly(DEAm-*co*-NEAm) show a deviation below the linear trend defined by the critical temperatures of the copolymers grafted with both homopolymers. In this case, one of the structural units of the copolymer is a secondary amide, whose nitrogen atom has two distinct possibilities for forming hydrogen bonds. This allows it to act simultaneously as both a donor and an acceptor in these intermolecular or intramolecular interactions. It suggests that, during the heating process, the formation of hydrophobic domains of *N*,*N*-diethylacrylamide units at mid-compositions could bring the NEAm and chitosan units closer together, favoring inter-chain hydrogen-bonding interactions and triggering the cooperative character of the transition [11]. Thus, for a given copolymer composition, rather than exhibiting an intermediate value for the transition temperature of both units, there is a decrease in the value of this parameter. Keerl and Richtering have also reported this type of behavior for hydrogels made from NIPAm and DEAm copolymers, which is consistent with the fact that these copolymers are composed by two types of *N*-alkyl-substituted units—one with a secondary amide nitrogen (NIPAm) and the other with a tertiary amide nitrogen (DEAm) [18]. Although the role of amide nitrogen in hydrogen-bonding interactions in hydrogels made from this type of copolymer is plausible, the effect of the macromolecular architecture and the distribution pattern of both structural units along the polymer chain cannot be ignored. This steric effect undoubtedly facilitates or hinders the formation of hydrogen bonds and ultimately affects the thermal transition [19].

Liu et al. reported similar findings in aqueous solutions of poly(DEAm-*co*-DMAm) and poly(DEAm-*co*-NEAm), where the addition of DMAm or NEAm units increased the copolymer hydrophilicity and elevated the *Tc*. For poly(DEAm-*co*-DMAm), however, the *Tc* curve followed a concave shape rather than a linear one with increasing DMAm content [14]. In aqueous solutions of poly(NIPAm-*co*-DMAm), a linear trend in *Tc* with increasing content of DMAm was observed [20]. Interestingly, the *Tc* of poly(NIPAm-*co*-DMAm) microgels synthesized using two different methods followed a linear or a non-linear trend, depending on the synthesis method used [21]. The latter seems to confirm the role of the macromolecular architecture and the distribution pattern of both structural units along the copolymer chain, which may or may not facilitate hydrogen-bond formation.

As explained above, when fitting the volume phase transition to the mathematical model, it is possible to estimate the *α* coefficient, which provides an indication of the transition slope and, consequently, its width. In physical terms, as *α* takes values greater than 0, the transition becomes smoother and more continuous.

**Figure 3:**
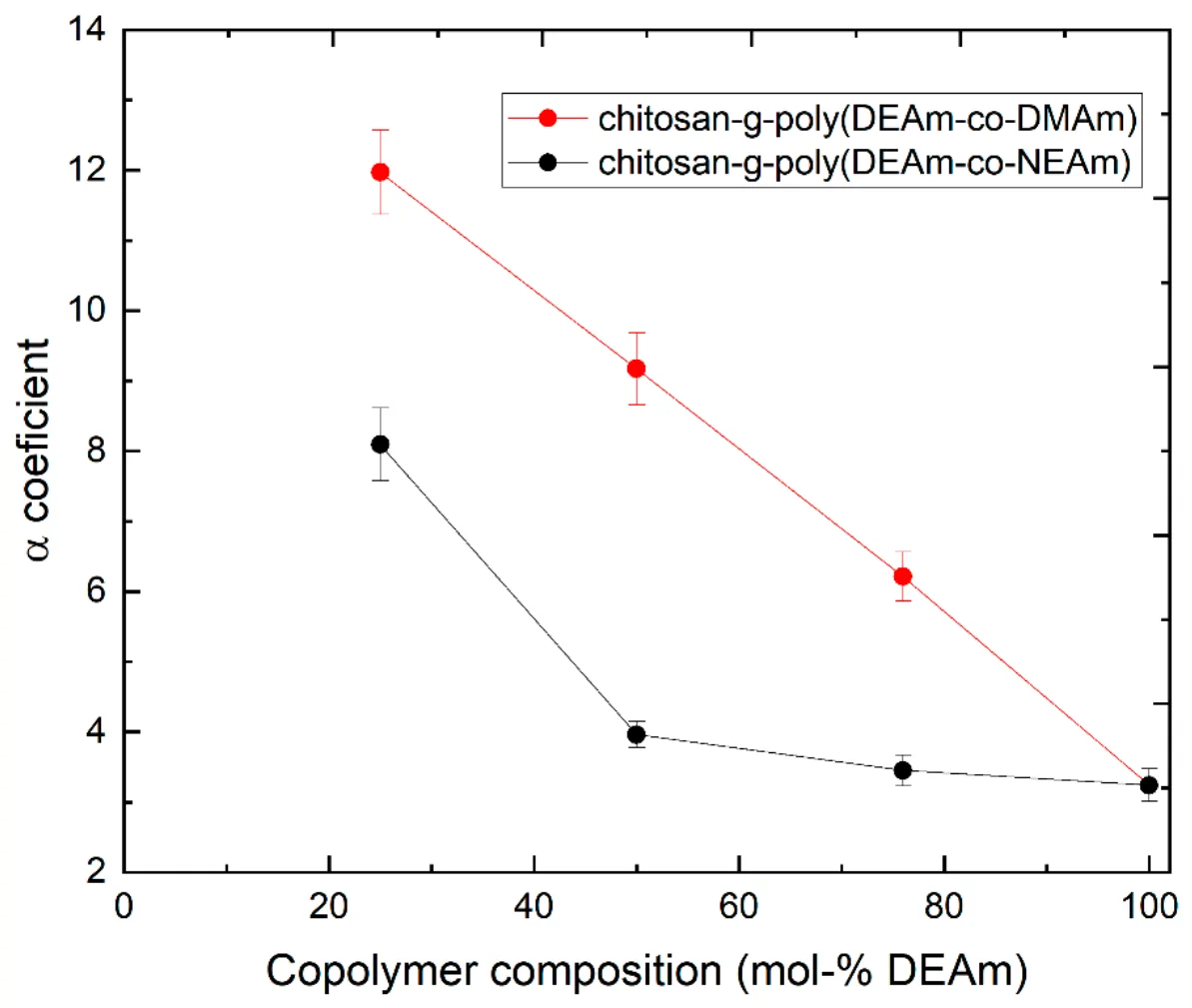
Composition dependence of *α* coefficient calculated from Equation (2) for the volume phase transition of chitosan-*g*-poly(DEAm-*co*-DMAm) and chitosan-*g*-poly(DEAm-*co*-NEAm) hydrogels.

The values of this coefficient are plotted in Figure 3 as a function of the composition of both copolymers. It is interesting to note that, in the case of the hydrogel made from the copolymer with DMAm, there is indeed a linear relationship with the proportion of hydrophilic units, similar to that observed for *Tc*. Once again, NEAm copolymers show a tendency to deviate from a linear relationship between the two structural units.

The molecular mechanism underlying the expansion of the volume phase transition is not yet fully understood. However, it is possible to speculate that, with hydrogen bonds playing a more prominent role, the contraction of the polymer chains occurs over a wider temperature range, possibly facilitated by a greater proximity between macromolecular chains, which allows for a more continuous transition starting at lower temperatures. Undoubtedly, the role of the balance between hydrophilic and hydrophobic units and the proportion of these two units in the copolymer must always be considered.

**Figure 4:**
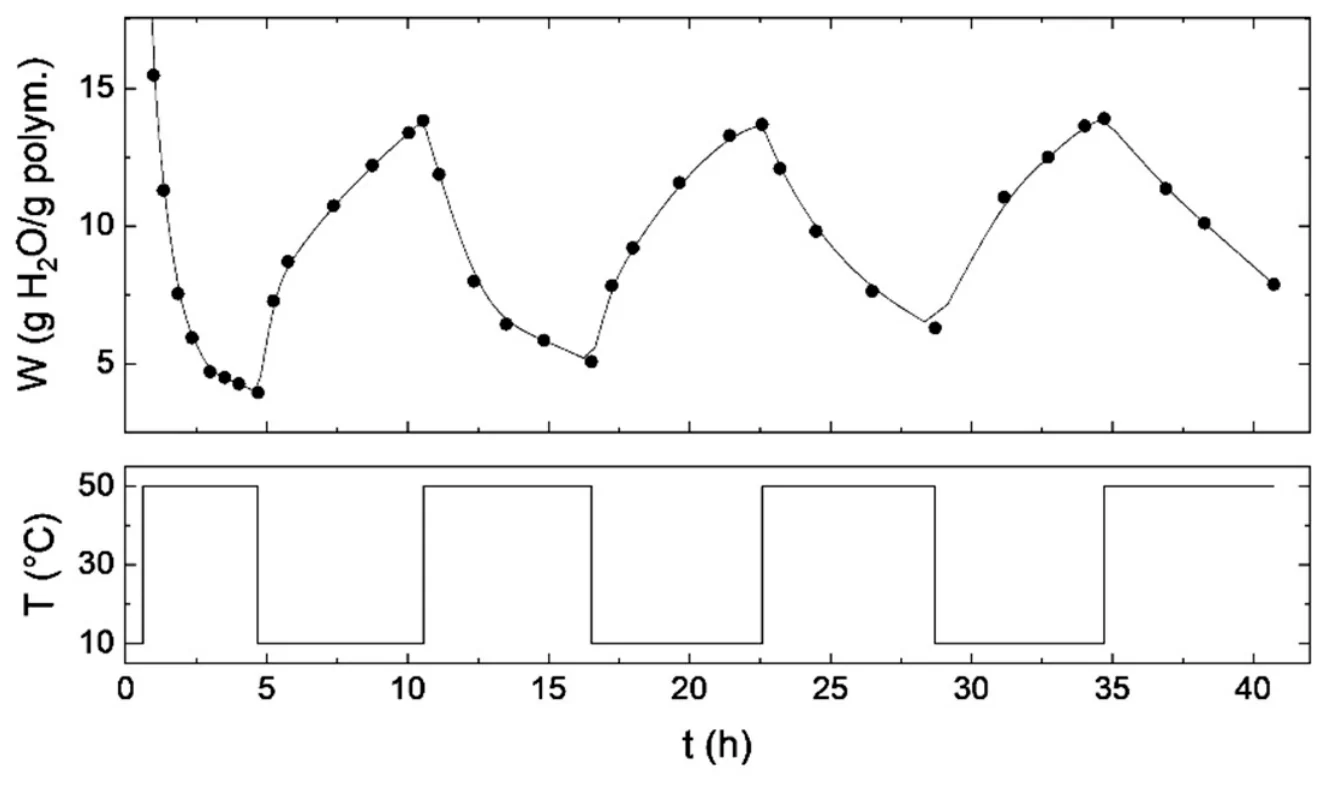
Thermoreversibility of the phase transition in chitosan-*g*-poly(DEAm-*co*-NEAm) 50 mol-% DEAm hydrogels. Black symbols represent the experimental data points, and the line is a visual guide.

The thermoreversibility of the hydrogel prepared from the chitosan-*g*-poly(DEAm-*co*-NEAm) 50 mol-% DEAm copolymer was determined by subjecting it to three continuous swelling and deswelling cycles. Figure 4 shows the swelling values measured over six-hour periods where the temperature was switched between 10 and 50 °C. This hydrogel shows a thermoreversible swelling behavior, and although the hydrogel response is not instantaneous—and periods of 6 h were apparently insufficient to reach equilibrium—there is no doubt that the material responds to temperature changes, with little “residual” change in the swelling values. After three successive cooling–heating cycles, no decrease in the maximum swelling values at 10 °C is apparent, but at 50 °C the minimum swelling value of the contracted polymer network is slightly increased. This is partly due to the incomplete recovery of the material under the experimental conditions used, as well as to its structural rigidity. This phenomenon is similar to what occurs in non-crosslinked elastomers, in which physical entanglements and chain free ends relax over long deformation times without returning to their original dimensions [22,23].

### 3.3. pH Response of Hydrogels

After evaluating the swelling and thermal behavior of these membranes in water, the pH response was also analyzed. This is an important characteristic because the presence of chitosan in the hydrogel not only provides it with good mechanical properties but also makes this material responsive to pH changes. Furthermore its polyelectrolyte behavior could affect its interactions with other chemical species and the factors that regulate the thermosensitive response of the material.

**Figure 5:**
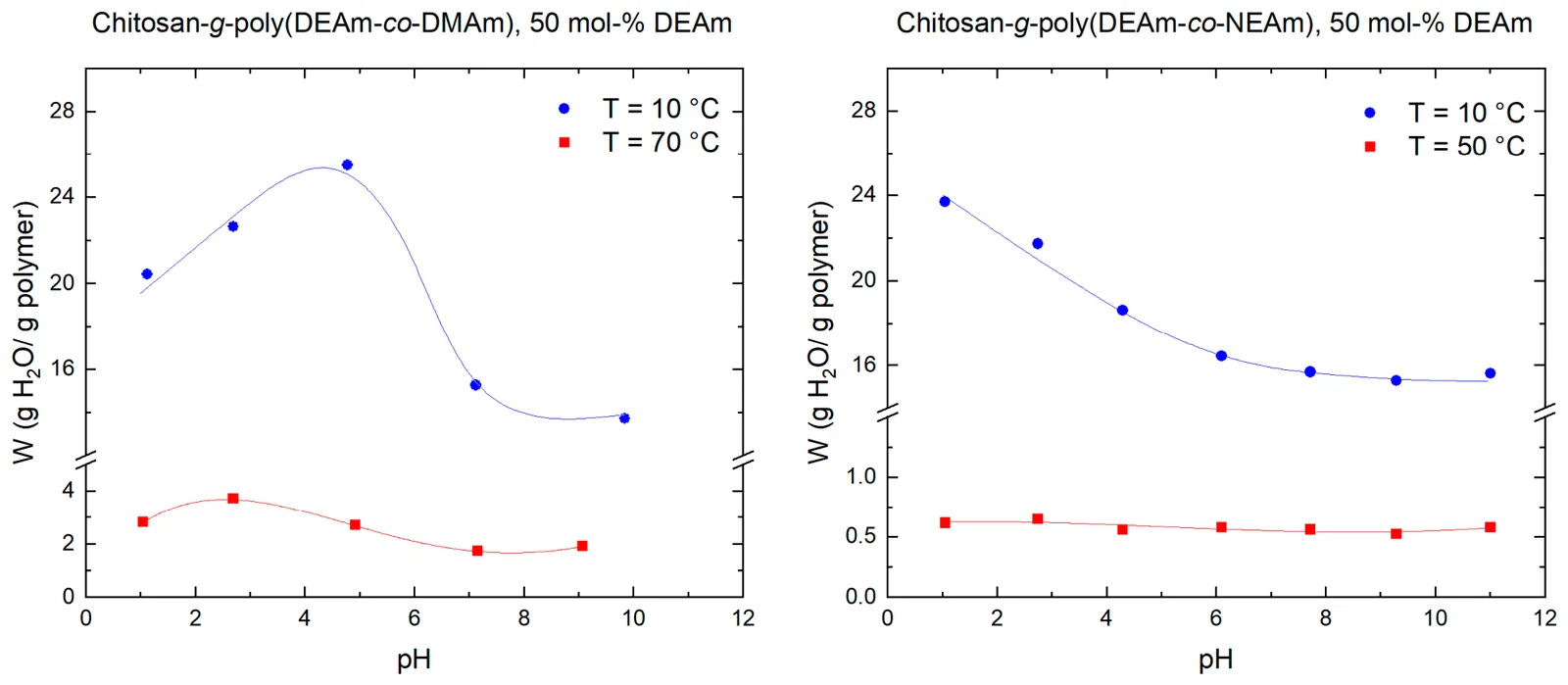
pH-dependent equilibrium swelling curves for chitosan-*g*-poly(DEAm-*co*-DMAm) and chitosan-*g*-poly(DEAm-*co*-NEAm) hydrogels at a composition of 50 mol-%.

Figure 5 shows the pH-dependent swelling response of both chitosan-*g*-poly(DEAm-*co*-DMAm) and chitosan-*g*-poly(DEAm-*co*-NEAm) hydrogels at a composition of 50 mol-% DEAm. Measurements were made at 10 °C and at 50 or 70 °C for the copolymer containing NEAm or DMAm, respectively. These two temperatures were selected to evaluate the pH response at a temperature well above the transition temperature.

At 10 °C (blue lines in Figure 5), the maximum swelling capacity for both hydrogels was achieved in the acidic region, driven by the protonation of the amino groups along the chitosan chains. Specifically, the copolymer containing DMAm reached its maximum swelling at pH 5, whereas the copolymer with NEAm achieved maximum swelling at pH 1. The electrostatic repulsion among the ionized groups fosters the expansion of the polymer network, thereby enabling higher swelling values. At extremely acidic pH, an enhancement in the ionic strength could decrease swelling by screening positive charges in chitosan-*g*-poly(DEAm-*co*-DMAm) hydrogels. Meanwhile, the increase in pH results in a decrease in swelling. As the $-{NH}_{3}^{+}$ groups become deprotonated, the macromolecular segments approach each other, resulting in the contraction of the polymer network.

Extensive deswelling occurred in both samples at temperatures above the *Tc*, regardless of the pH value (red curves in Figure 5). This effect is widely understood to be responsible for the extremely low swelling values observed under hot conditions. This is especially true for the NEAm copolymer, for which almost no variation in swelling with increasing pH at 50 °C is observed. This behavior suggests that—at high temperatures—polymer-polymer hydrophobic interactions prevail and even overcome the electrostatic repulsive forces from chitosan chains in acidic media.

Similar effects were previously observed in chitosan-*g*-poly(NIPAm)/pectin polyelectrolyte complex membranes [24], although the authors reported a less pronounced deswelling behavior. This discrepancy could be related to a lower amount of thermosensitive polymer (approximately 26%) in their materials compared to ours (~83%). Under strongly basic conditions and high temperatures, hydrolysis of *N*-alkyl acrylamide groups is more likely to occur, leading to the degradation of hydrogels. Accordingly, these samples were not considered.

### 3.4. Methylene Blue Release Experiments

Finally, experiments were carried out to release a model substance (methylene blue) from the hydrogel. These trials were conducted in water, at two temperatures, below (10 °C) and above (50 °C) the volume phase transition. When dry hydrogels loaded with a solute come into contact with water (which in this case is the release medium) at a constant temperature, the solute diffuses out of the hydrogel, and the hydrogel swells due to an influx of water.

**Figure 6:**
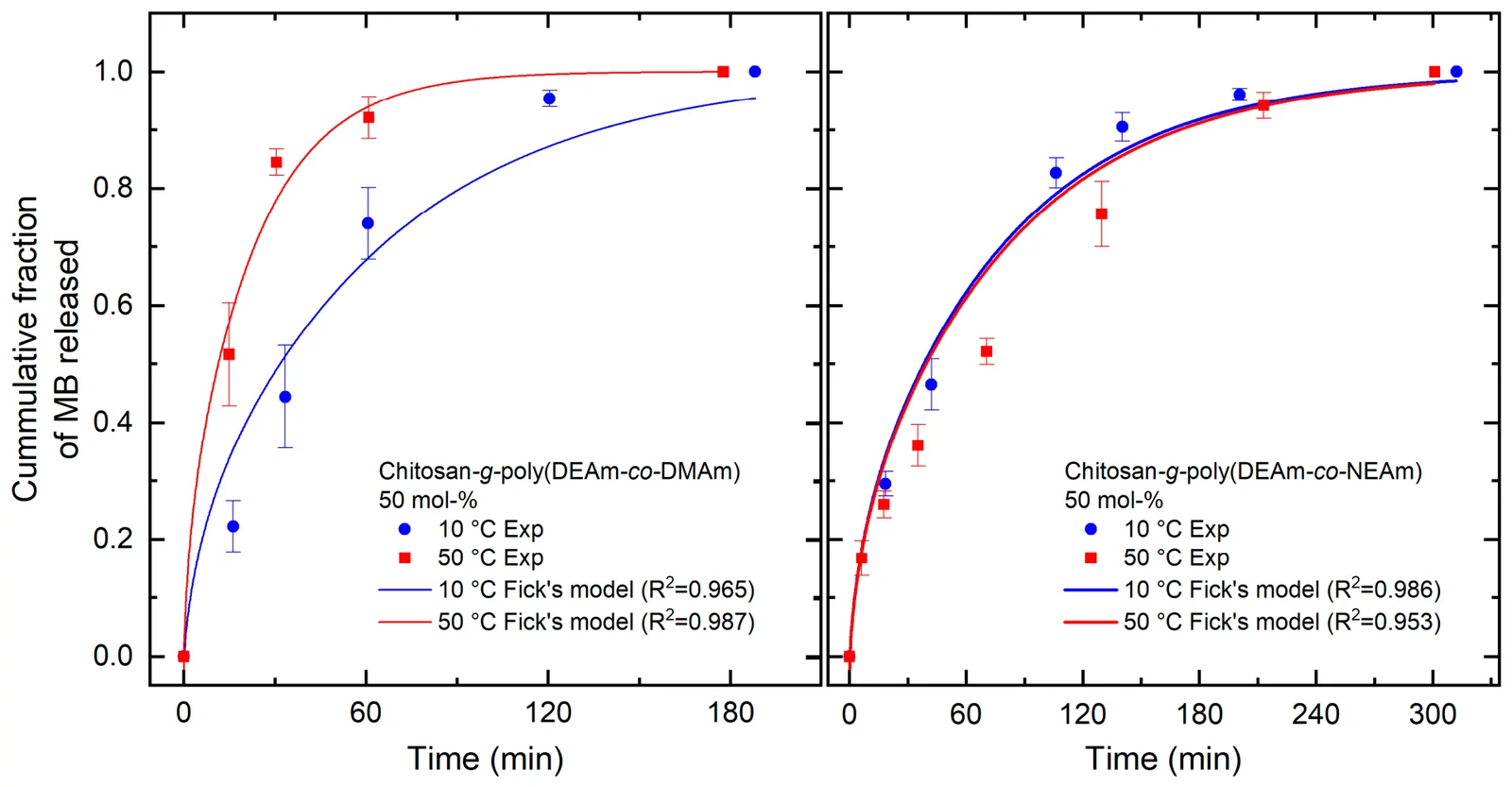
Cumulative fraction of methylene blue released in water (pH 6) from chitosan-*g*-poly(DEAm-*co*-DMAm) and chitosan-*g*-poly(DEAm-*co*-NEAm) hydrogels at a 50 mol-% DEAm composition.

Figure 6 shows the release profiles of methylene blue (MB) from chitosan-*g*-poly(DEAm-*co*-DMAm) and chitosan-*g*-poly(DEAm-*co*-DMAm) 50 mol-% DEAm hydrogels. The solute was assumed to diffuse through the hydrogels towards the solvent according to Fick’s diffusion law. The implemented model solves Fick’s second law of diffusion for one-dimensional diffusion during the desorption of a solute from a 2*l* thick membrane. In other words, it is assumed that the substance diffuses from the parallel planes and only an insignificant amount moves radially. An initially uniform concentration of the substance is considered inside the hydrogel, and a time-dependent concentration, *C_t_*, in the supernatant. Equation (3) can be used as a rough approximation of these boundary conditions to estimate the diffusion coefficient for comparative purposes.

The model equation can be written as [25]:

(3) $$\frac{C_{t}}{C_{max}}=1-\sum_{n=0}^{\infty }\frac{8}{{\left(2n+1\right)}^{2}\pi^{2}}e^{\left(-\frac{D{\left(2n+1\right)}^{2}\pi^{2}}{4l^{2}}\right)t}$$

Equation (3) was solved for the first 16 terms of the summation to fit the experimental data using the non-linear least squares method by adjusting the ratio *D*/*l*^2^. *D* is the diffusion coefficient of the substance desorbing from the hydrogel. In all cases, the ANOVA gave *p* values < 1 × 10^−4^.

Figure 6 shows the normalized release curves according to Equation (3). The MB release profiles of the two hydrogel samples were different. It is notable that while the NEAm hydrogel exhibits a similar behavior at both temperatures, the copolymer with DEAm shows a different pattern. In the latter case, the release rate at 50 °C is significantly faster than at 10 °C. The release process for the chitosan-*g*-poly(DEAm-*co*-NEAm) hydrogel extends over longer periods of time. An explanation for this is not apparent at this time, but it is likely a consequence of differences in hydrogen bonding and/or differences in hydrophilicity between the monomer units of both copolymers.

The diffusion coefficient values calculated using Equation (3) are summarized in Table 1. These values are one order of magnitude lower than those theoretically estimated for MB diffusion in pure water, which range from 4.4 to 12 × 10^−10^ m^2^·s^−1^ at 20 and 50 °C, respectively, as estimated from the Stokes–Einstein dependence D∝T/η [26,27].

The solute diffusion is the overall result of complex diffusion processes taking place simultaneously. On the one hand, there is the swelling of the polymer matrix, which results in a relaxation of polymer chains and an increase in the diffusion paths across the hydrogel. On the other hand, the solute is dissolved in the solvent (water in this case) and begins to diffuse. The observed release behavior is the result of these phenomena, leading to an anomalous diffusion pattern. The diffusion coefficient values are one order of magnitude lower than in free water, which supports the aforementioned discussion.

## 4. Conclusions

The hydrogels based on chitosan grafted with copolymers of poly(DEAm) and one of the two more hydrophilic units, *N*,*N*-dimethylacrylamide (DMAm) or *N*-ethylacrylamide (NEAm), at various compositions were successfully prepared through radical polymerization.

All thermosensitive hydrogels exhibited a continuous volume phase transition that broadened with increasing content of the hydrophilic units (DMAm and NEAm). The critical temperatures of the chitosan-*g*-poly(DEAm-*co*-DMAm) hydrogels increased linearly with the content of DMAm units in the copolymer. In chitosan-*g*-poly(DEAm-*co*-NEAm) hydrogels, *Tc* values followed a concave shape rather than a linear one with increasing content of NEAm. This indicated that the formation of hydrophobic domains of *N*,*N*-diethylacrylamide units during heating could bring the *N*-alkyl polyacrylamide and chitosan chains closer together, favoring inter-chain hydrogen bonding, thus promoting the cooperative character of the transition.

The pH response observed in both hydrogels was provided by chitosan. Acidic pH values expanded the polymer network and increased swelling due to the electrostatic repulsion among the protonated amino groups along the chitosan chains. Hence, this expansion diminished as the pH became more basic. Hydrogel shrinking due to the phase transition almost canceled the pH response above the critical temperature.

## Figures and Tables

**Table 1:** Diffusion coefficients of methylene blue released in water (pH 6) and estimated using Equation (3).

Hydrogel Sample	T	D × 10^11^
	°C	m^2^ s^−1^
Chitosan-*g*-poly(DEAm-*co*-DMAm) 50 mol-% DEAm	10	7.5
	50	20.8
Chitosan-*g*-poly(DEAm-*co*-NEAm) 50 mol-% DEAm	10	6.2
	50	5.1

## Data Availability

The original contributions presented in this study are included in the article. Further inquiries can be directed to the corresponding authors.
